# Prevalence of pre-diabetes/type 2 diabetes among adolescents (10–19 years) and its association with different measures of overweight/obesity in India: a gendered perspective

**DOI:** 10.1186/s12902-021-00802-w

**Published:** 2021-07-07

**Authors:** Pradeep Kumar, Shobhit Srivastava, Prem Shankar Mishra, E. T. Krishnan Mooss

**Affiliations:** 1 International Institute for Population Sciences, Mumbai, Maharashtra 400088 India; 2 Institute for Social and Economic Change, Bengaluru, Karnataka 560072 India; 3 Vaidyaratmam Group, Thrissur, India

**Keywords:** Pre-diabetes/diabetes, Obesity, Adolescents, India

## Abstract

**Background:**

The International Diabetes Federation (IDF) estimated that 1.1 million children and adolescents aged 14–19 years are living with diabetes. Diabetes is a chronic, progressive disease characterized by elevated levels of blood glucose. It is also recognized as a complex disease that affects people of different ages due to different causes. The present study aims to estimate the prevalence of pre-diabetes/diabetes at the national level. Additionally, the respective study determines the factors associated with pre-diabetes/diabetes conditions among adolescents at the national level.

**Methods:**

The data for this study was carried out from the Comprehensive National Nutrition Survey (CNNS), the first-ever nationally representative nutrition survey of children and adolescents in India. The study used a sample size of 17,865 adolescent boys and 17,965 adolescent girls for the analysis. Descriptive statistics, bivariate analysis, and logistic regression analysis were done to carve out the results.

**Results:**

The prevalence of pre-diabetes/diabetes was 12.3% and 8.4% among adolescent boys and girls in India, respectively. Body mass index and Subscapular skinfold thickness were the two most important predictors of pre-diabetes/diabetes among adolescents. Further, physical activities show a negative association with pre-diabetes/diabetes. Moreover, interaction models in the present study clearly reveal the fact that adolescent girls were less likely to suffer from pre-diabetes/diabetes than adolescent boys. Additionally, it was found that the prevalence of pre-diabetes/diabetes was high among adolescent girls from lower socio-economic strata.

**Conclusion:**

The high prevalence of pre-diabetes and diabetes among adolescents portrayed serious public health concern in India. As body mass index and Subscapular skinfold thickness were positively associated with pre-diabetes/diabetes conditions among adolescents. Therefore, effective approaches are needed to be taken to tackle these pre-diabetes/diabetes conditions among adolescents and especially among adolescent boys.

## Background

The International Diabetes Federation (IDF) estimated that 1.1 million children and adolescents aged 14–19 years were living with diabetes [1]. In addition, the increasing prevalence of pre-diabetes and diabetes led by obesity among the young population fuelled metabolic syndrome and obesity-related co-morbidities across the low and middle-income countries [2]. There is a global effort to curb down the rise of diabetes and obesity by 2025; however, a steady rise in the prevalence of diabetes is a matter of concern [3].

India alone contributes a larger proportion to premature deaths from non-communicable diseases (NCDs), including diabetes across all socio-economic groups [4]. Achieving the global agenda of Sustainable Development Goals (SDGs) by 2030, by reducing one-third of premature mortality from NCDs–including diabetes, a universal healthcare access approach is therefore required in most developing countries, including India [3, 4]. India is currently going through the nutritional transition, and it is evident with a rise in the prevalence of overweight and obesity [5]. Also, estimates shows that nearly 166 million adults are overweight/obese in 2016 in India [3]. In the case of adults who are affected by diabetes, the numbers are estimated to be nearly 73 million, the second-largest in the world [1].

Diabetes is a chronic, progressive disease characterized by elevated levels of blood glucose levels. It is also recognized as a complex disease that affects people of different ages due to different causes. There are three main types of diabetes, type-1 diabetes (T1D), type-2 diabetes (T2D), and gestational diabetes mellitus (GDM) [3]. The type-1 diabetes incidence rate is increasing among the younger population, and the contributing factors have remained unclear. However, there are factors that affect the heterogeneity of the respective disease such as genetic factors, behavioural risk factors, food and dietary habits, physical inactivity, lifestyle changes.

Furthermore, the prevalence of pre-diabetes/diabetes among children, adolescents, and even younger adults is increasing at a much higher rate irrespective of socio-economic strata, and it becomes more severe because of the rising levels of obesity among them [6–8], physical inactivity [9, 10], and poor dietary habits [11]. Moreover, increased levels of substance use [12, 13] among adolescent groups have also led to risk for pre-diabetes/diabetes-associated health issues [10, 14]. Further, the high spread of pre-diabetes/diabetes among the population is also witnessed in the light of different socio-economic groups [15], and it is concentrated differently across rural-urban, age-sex, and family history of diabetes [16–18].

However, lack of physical activities and having a sedentary lifestyle among adolescents made responsible for cardiovascular disease and obesity-related risk factors which in turn may cause menace of pre-diabetes/diabetes among them [10, 19, 20]. That has not been well established in the literature in the Indian context among the adolescents population. In this way, there are multiple determinants associated with risk factors for pre-diabetes and diabetes that influence the adolescent population and they are more prone to be affected by them [16, 21]. Moreover, Triglycerides, Cholesterol, High-density lipoprotein (HDL), and Low-density lipoprotein (LDL) levels are found to be significant factors for the prevalence of pre-diabetes/diabetes among adolescents [22, 23].

The present study utilized the different forms of obesity, which may affect the chaos of pre-diabetes/diabetes differently. Previous literature showed different measures of obesity in their studies [11, 24–26]. For instance, obesity measured through body mass index [23], waist circumference [23], waist to hip ratio [27], Subscapular skinfold thickness (SSFT) [28] and Triceps skinfold thickness (TSFT) [28].

There exists a dearth of literature focusing on the association of pre-diabetes/diabetes with obesity levels among adolescents at the national level in India. Therefore, the present study aims to estimate the prevalence of pre-diabetes/diabetes at the national level. Additionally, the study also analyses the factors that are responsible for pre-diabetes/diabetes conditions among adolescents (for both adolescent boys and girls) at the national level. The study hypothesized that there was no association between pre-diabetes/diabetes conditions with obesity levels and physical activity among adolescents in India.

## Methods

The data was taken from the Comprehensive National Nutrition Survey (CNNS), the first-ever nationally representative nutrition survey of children and adolescents in India. The survey collected the data on the status of pre-school (0–4 years), school-age children (5–9 years), and adolescents (10–19 years) through interviews, a comprehensive set of anthropometric measures, and biochemical indicators. The CNNS covered all 30 states of India using a multi-stage survey design for the selection of households and individuals aged 0–19 years. The data has been collected with the written consent of the participants. The survey was implemented under the guidance of the MoHFW, UNICEF, a Technical Advisory Group (TAG), and the Center for Disease Control (CDC). Data can be obtained on request from the Population Council (India office) website. The detailed methodology, sampling design, and data collection procedure were published in the survey report [29]. The CNNS collected data from a total of 38,060 children aged 0–4 years, 38,355 children aged 5–9 years, and 35,830 adolescents age 10–19 years. The study used a sample size of 17,865 adolescent boys and 17,965 adolescent girls for the analysis.

### Outcome variable

The outcome variable was pre-diabetes/diabetes conditions among adolescents aged 10–19 years. The pre-diabetes/diabetes was diagnosed using glycosylated haemoglobin (HbA1c) [30]. The American Diabetes Association recommended glycated hemoglobin (HbA1c) as a possible substitute to fasting blood glucose for diagnosis of diabetes [31, 32]. Analysis of glycated hemoglobin (HbA1c) in blood provides the evidence about the respondent’s/individual average blood glucose levels during the last 2–3 months, which is the predicted half-life of red blood cells (RBCs) [33]. The cut-off points for pre-diabetes/diabetes among adolescents were as: pre-diabetes (> 5.8% and ≤ 6.4%) and diabetes (> 6.4%) [34]. We merged diabetes and pre-diabetes into one category due to the small sample in the diabetes condition. The outcome variable was divided into two categories as 0 “no pre-diabetes/diabetes” and 1 “pre-diabetes/diabetes”. The type of diabetes studied is Type 2 Diabetes mellitus. The study did not include fasting glucose data as HbA1c reflects average plasma glucose levels over the previous 8 to 12 weeks [28].

### Exposure variables

Body mass index was categorized as not overweight/obese (BMI ≤ + 1 SD) and overweight/obese (BMI > + 1 SD) group, abdominal obesity was categorized as no (waist circumference-for-age ≤ + 1 SD) and yes (waist circumference-for-age > + 1 SD), Triceps skinfold thickness (TSFT) was grouped as not overweight (TSFT-for-age ≤ + 1 SD) and overweight (TSFT-for-age > + 1 SD) and Subscapular skinfold thickness (SSFT) was divided into two categories: not overweight (SSFT-for-age ≤ + 1 SD) and overweight (SSFT-for-age > + 1 SD). Body mass index *z*-scores, also called BMI standard deviation (s.d.) scores, are measures of relative weight adjusted for child age and sex [35]. Given a child’s age, sex, BMI, and an appropriate reference standard, a BMI *z*-score (or its equivalent BMI-for-age percentile) can be determined [35]. Physical activity was categorized as a low, medium, and high using the principal component analysis approach as 15 questions were used to generate the respective variable [29]. Serum triglycerides (assessed by spectrophotometry and enzymatic endpoint method) was categorized into low (< 130 mg/dl) and high (≥130 mg/dl), cholesterol (assessed by spectrophotometry using cholesterol oxidase esterase peroxidase) was recoded as low (< 200 mg/dl) and high (≥200 mg/dl), low-density lipoprotein (LDL) (assessed by spectrophotometry and direct measure cholesterol oxidase) was categorized as low (< 130 mg/dl) and high (≥130 mg/dl), and high-density lipoprotein (HDL) cholesterol (assessed by spectrophotometry and direct measure polyethylene glycol-modified cholesterol oxidase) was recoded as low (< 40 mg/dl) and high (≥40 mg/dl). Age was categorized into early adolescents (10–14 years) and late adolescents (15–19 years), education was divided into no schooling, dropouts and currently studying, Media exposure was categorized as no “no exposure to television & radio & newspaper & internet” and yes “exposure to television or radio or newspaper or internet), parent education was recoded as (both uneducated, anyone educated and both educated), parent diabetes status was categorized as (no or anyone had), the diet was categorized as vegetarian and non-vegetarian; Junk food consumption was categorized as no, 1–2 days and 3–7 days; substance use was categorized as no and yes. Caste was categorized as Scheduled Caste/Scheduled Tribe (SC/ST) and non- Scheduled Caste/Scheduled Tribe. Among the four categories of castes, people belonging to the Scheduled caste lies at the bottom of the Indian caste system and have been exploited for over centuries. The indigenous groups of India belong to the category of Scheduled tribes and are among the most deprived sections. Therefore SC/ST is the deprived section of the society [36]. Religion was categorized as Hindu, Muslim, Christian, and Others; wealth quintile was categorized as poor, middle and rich; the residence was categorized as urban and rural; and region were divided as north, central, east, north-east, west, and south.

### Statistical analysis

Descriptive analysis was used to show the sample profile of the respondents. Further, bivariate analysis was carried out to estimate the prevalence of pre-diabetes/diabetes by selected variables, and a proportion test was applied to test the significant difference between adolescent boys and girls for pre-diabetes/diabetes. Finally, multivariate logistic regression was performed to find out the effects of different predictors on pre-diabetes/diabetes. The results are presented in the form of an odds ratio (OR) with a 95% confidence interval (CI). The model is usually put into a more compact form as follows:

$$ \ln \left(\frac{P_i}{1-{P}_i}\right)={\beta}_0+{\beta}_1{x}_1+\dots +{\beta}_M{x}_M, $$

Where *β*_0_, …. . , *β*_*M*_ are the regression coefficient indicating the relative effect of a particular explanatory variable on the outcome, the study further examined the possible interaction between body mass index, abdominal obesity, triceps skinfold thickness (TSFT), subscapular skinfold thickness (SSFT), and gender in models 2, 3, 4, and 5 respectively.

## Results

Anthropometric and socio-demographic profiles of adolescents aged 10–19 years are presented in Table 1. Overall, nearly 5% of adolescent boys and girls (each) were overweight/obese whereas about 2% and 1% of adolescent boys and girls had abdominal obesity, respectively. Moreover, about 5% and 2% of adolescent boys and girls had triceps skinfold thickness, respectively. Seven percent of adolescent boys and 4% of girls had subscapular skinfold thickness. About 14% of boys and 18% of adolescent girls had high serum triglycerides. Moreover, almost the same proportion of adolescents (3% vs. 5%) had high cholesterol and low-density lipoprotein respectively. The proportion of adolescents was higher in the 10–14 years age group irrespective of gender. The majority of adolescents are currently studying and, around 17% of adolescent boys and 21% of girls were school dropouts. More than three-fourth of adolescents had some mass media exposure. One-fourth of the parents had no schooling and a very low proportion of parents (anyone) had diabetes (4%). About 81% of adolescent boys and 73% of girls were non-vegetarian. Seven out of ten adolescents were consumed 1–2 days of junk food**.** Substance use was higher among adolescent boys (8%) than girls. One-third of adolescents belonged to SC/ST group and the majority of adolescents were Hindu. Two-fifth of adolescents belonged to rich families and one-fourth of adolescents lived in urban areas.

**Table 1:** Anthropometric and socio-demographic profile of adolescents aged 10–19 years in India, 2016–18

Variables	Adolescent boys		Adolescent girls	
	Sample	Percent	Sample	Percent
**Body mass Index (BMI)**				
Not overweight/obese	15,697	95.0	14,841	95.1
Overweight/obese	821	5.0	761	4.9
**Abdominal obesity**				
No	17,116	97.8	16,271	98.8
Yes	381	2.2	202	1.2
**Triceps skinfold thickness (TSFT)**				
Not overweight	16,675	94.7	16,276	98.3
Overweight	937	5.3	276	1.7
**Subscapular skinfold thickness (SSFT)**				
Not overweight	16,356	92.9	15,826	95.7
Overweight	1249	7.1	704	4.3
**Physical activity**				
Low	6963	37.8	12,178	70.0
Medium	4975	27.0	2544	14.6
High	6486	35.2	2683	15.4
**Serum triglycerides**				
Low	5856	85.9	5241	81.7
High	963	14.1	1172	18.3
**Cholesterol**				
Low	6582	96.6	6112	95.4
High	234	3.4	293	4.6
**Low-density lipoprotein**				
Low	6591	96.8	6121	95.5
High	220	3.2	289	4.5
**High-density lipoprotein**				
Low	2008	29.6	1533	24.0
High	4780	70.4	4854	76.0
**Age (years)**				
Early adolescence (10–14)	9757	53.0	8832	50.7
Late adolescence (15–19)	8668	47.0	8573	49.3
**Education**				
No schooling	885	4.8	1182	6.8
Dropout	3194	17.3	3721	21.4
Currently studying	14,346	77.9	12,502	71.8
**Media exposure**				
No	2955	16.0	4080	23.4
Yes	15,470	84.0	13,325	76.6
**Parent education**				
Both uneducated	4487	24.4	4529	26.0
Anyone educated	5908	32.1	5782	33.2
Both educated	8030	43.6	7094	40.8
**Parents diabetes status**				
No one	17,675	95.9	16,769	96.4
Anyone had	750	4.1	636	3.7
**Diet**				
Vegetarian	3519	19.1	4770	27.4
Non-vegetarian	14,902	80.9	12,634	72.6
**Junk food consumption**				
No	4876	26.5	4638	26.7
1–2 days	12,771	69.3	12,216	70.2
3–7 days	774	4.2	549	3.2
**Substance use**				
No	16,876	91.6	17,194	98.8
Yes	1549	8.4	211	1.2
**Caste**				
SC/ST	5769	33.2	5769	33.2
Non-SC/ST	11,636	66.9	11,636	66.9
**Religion**				
Hindu	14,878	80.8	13,797	79.3
Muslim	2643	14.3	2819	16.2
Christian	458	2.5	381	2.2
Others	446	2.4	408	2.3
**Wealth quintile**				
Poor	7292	39.6	7036	40.4
Middle	3696	20.1	3473	20.0
Rich	7437	40.4	6896	39.6
**Place of residence**				
Rural	13,788	74.8	13,174	75.7
Urban	4637	25.2	4231	24.3
**Regions**				
North	2597	14.1	2306	13.3
Central	5750	31.2	5936	34.1
East	4248	23.1	4148	23.8
Northeast	582	3.2	507	2.9
West	2327	12.6	1864	10.7
South	2921	15.9	2644	15.2
**Total**	17,865	100.0	17,965	100.0

*SC/ST* Scheduled caste/ Scheduled tribe

**Fig. 1:**
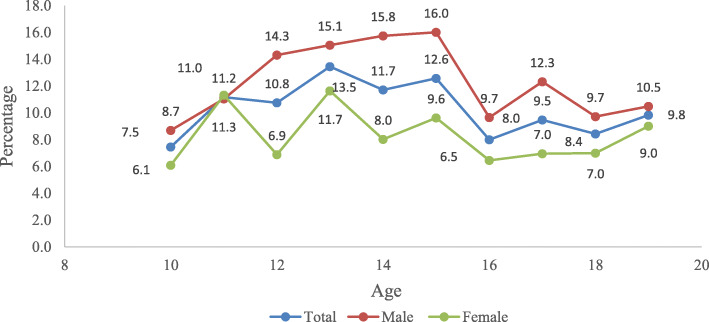
Gender differentials for pre-diabetes/diabetes among adolescents in India

Gender differentials for pre-diabetes/diabetes among adolescents are presented in Fig. 1. Pre-diabetes/diabetes was more prevalent among 15 years of adolescents, and it is lower in 10 years of adolescents. Though, the prevalence of pre-diabetes/diabetes was higher among adolescent boys compared to girls irrespective of their age.

Table 2 shows the gender differences in the prevalence of pre-diabetes/diabetes among adolescents aged 10–19 years across different socio-demographic characteristics in India. The prevalence of pre-diabetes/diabetes was higher among adolescent boys across all the selected socio-demographic characteristics and differences were statistically significant. Substantial differences were observed among adolescents who had abdominal obesity (11.7%), overweight (SSFT) (9.8%), belonged to the Northeast region (10.1%), consumed junk food for 3–7 days (8.5%), and belonged to the middle (6.4%) and rich families (5.6%).

**Table 2:** Prevalence of pre-diabetes/diabetes among adolescents aged 10–19 years by socio-demographic characteristics in India, 2016–18

Variables	Adolescent boys	Adolescent girls	Adolescent boys - Adolescent girls	Proportion Test
	(%)	(%)	(%)	***P*** < 0.05
**Body Mass Index (BMI)**				
Not overweight/obese	12.1	8.2	3.9	
Overweight/obese	17.2	11.5	5.7	*
**Abdominal obesity**				
No	12.2	8.3	3.8	*
Yes	20.8	9.1	11.7	
**Triceps skinfold thickness (TSFT)**				
Not overweight	12.0	8.3	3.7	
Overweight	18.6	10.5	8.0	
**Subscapular skinfold thickness (SSFT)**				
Not overweight	11.6	8.1	3.5	
Overweight	22.6	12.8	9.8	*
**Physical activity**				
Low	13.6	8.3	5.4	*
Medium	12.2	9.2	3.0	*
High	11.6	7.5	4.0	*
**Serum triglycerides**				
Low	12.2	9.2	3.0	*
High	10.3	5.5	4.8	*
**Cholesterol**				
Low	12.0	8.5	3.5	*
High	9.1	8.5	0.6	*
**Low-density lipoprotein**				
Low	11.9	8.3	3.6	*
High	13.2	13.1	0.1	*
**High-density lipoprotein**				
Low	8.7	7.9	0.8	*
High	12.3	8.7	3.7	*
**Age (years)**				
Early adolescence (10–14)	13.0	8.7	4.3	*
Late adolescence (15–19)	11.7	7.8	3.9	*
**Education**				
No schooling	8.2	7.7	0.5	
Dropout	12.3	9.4	2.9	*
Currently studying	12.6	8.1	4.6	*
**Media exposure**				
No	11.8	7.1	4.7	
Yes	12.6	8.6	4.0	*
**Parent education**				
Both uneducated	12.5	8.0	4.5	*
Anyone educated	12.7	9.3	3.4	*
Both educated	12.3	7.7	4.6	*
**Parents diabetes status**				
No one	12.5	8.4	4.2	*
Anyone had	10.2	6.7	3.5	
**Diet**			0.0	
Vegetarian	14.1	10.3	3.9	*
Non-vegetarian	12.0	7.6	4.5	*
**Junk food consumption**				
No	12.9	10.7	2.2	*
1–2 days	12.1	7.5	4.7	*
3–7 days	14.1	5.7	8.5	*
**Substance use**				
No	11.9	8.2	3.7	*
Yes	18.9	14.1	4.8	*
**Caste**				
SC/ST	11.8	9.5	2.4	*
Non-SC/ST	12.7	7.7	5.0	*
**Religion**				
Hindu	12.3	8.5	3.8	*
Muslim	11.8	6.3	5.5	*
Christian	18.1	12.3	5.8	*
Others	15.3	10.1	5.1	
**Wealth quintile**				
Poor	10.8	9.4	1.4	*
Middle	14.0	7.6	6.4	*
Rich	13.3	7.6	5.6	*
**Place of residence**				
Rural	12.4	8.4	4.0	*
Urban	12.7	8.0	4.7	*
**Regions**				
North	16.3	10.6	5.8	*
Central	11.1	6.9	4.2	*
East	9.3	5.5	3.7	*
Northeast	18.4	8.3	10.1	*
West	13.7	12.9	0.9	
South	14.5	10.2	4.3	*
**Total**	12.4	8.3	4.1	*

*if *p* < 0.05; *SC/ST* Scheduled caste/ Scheduled tribe, *%* Percentage

Binary logistic regression estimates for pre-diabetes/diabetes among adolescents aged 10–19 years are presented in Table 3*.* Results from model-1 found that overweight/obese adolescents had 41% higher chances [Odd ratio (OR), 1.41; CI: 1.08–1.82] to get pre-diabetes/diabetes compared to adolescents who were not overweight/obese. Moreover, SSFT had a significant effect on pre-diabetes/diabetes among adolescents. Overweight adolescents (SSFT) were significantly more likely (OR, 1.49: CI: 1.15–1.94) to suffer from pre-diabetes/diabetes than those who were not overweight. There was a negative relationship between the physical activity of adolescents and their pre-diabetes/diabetes condition. Adolescents involved in high physical activity were less likely to suffer from pre-diabetes/diabetes compared to those who did low physical activities. However, the result was not significant for physical activities.

**Table 3:** Logistic regression estimates for pre-diabetes/diabetes among adolescents aged 10–19 by socio-demographic characteristics in India, 2016–18

Variables	Model-1	Model-2	Model-3	Model-4	Model-5
	OR(95% CI)	OR(95% CI)	OR(95% CI)	OR(95% CI)	OR(95% CI)
**Body mass index (BMI)**					
Not overweight/obese	Ref.		Ref.	Ref.	Ref.
Overweight/obese	1.41*(1.08,1.82)		1.4*(1.08,1.82)	1.39*(1.07,1.81)	1.39*(1.07,1.81)
**Abdominal obesity**					
No	Ref.	Ref.		Ref.	Ref.
Yes	1.06 (0.73,1.54)	1.09 (0.75,1.59)		1.07 (0.73,1.55)	1.07 (0.74,1.56)
**Triceps skinfold thickness (TSFT)**					
Not overweight	Ref.	Ref.	Ref.		Ref.
Overweight	0.89 (0.64,1.24)	0.92 (0.66,1.27)	0.9 (0.65,1.25)		0.92 (0.66,1.28)
**Subscapular skinfold thickness (SSFT)**					
Not overweight	Ref.	Ref.	Ref.	Ref.	
Overweight	1.49*(1.15,1.94)	1.49*(1.15,1.94)	1.5*(1.15,1.95)	1.5*(1.15,1.95)	
**Physical activity**					
Low	Ref.	Ref.	Ref.	Ref.	Ref.
Medium	0.90 (0.76,1.06)	0.90 (0.76,1.06)	0.90 (0.76,1.06)	0.90 (0.76,1.06)	0.90 (0.76,1.06)
High	0.80 (0.85,1.14)	0.80 (0.85,1.14)	0.80 (0.85,1.14)	0.80 (0.84,1.14)	0.80 (0.85,1.14)
**Serum triglycerides**					
Low	Ref.	Ref.	Ref.	Ref.	Ref.
High	0.90 (0.75,1.07)	0.90 (0.75,1.07)	0.90 (0.75,1.07)	0.90 (0.76,1.07)	0.90 (0.75,1.07)
**Cholesterol**					
Low	Ref.	Ref.	Ref.	Ref.	Ref.
High	1.02 (0.69,1.52)	1.03 (0.69,1.53)	1.03 (0.69,1.52)	1.03 (0.69,1.53)	1.03 (0.69,1.53)
**Low-density lipoprotein**					
Low	Ref.	Ref.	Ref.	Ref.	Ref.
High	1.33 (0.93,1.9)	1.32 (0.92,1.9)	1.33 (0.93,1.9)	1.33 (0.93,1.91)	1.33 (0.92,1.9)
**High-density lipoprotein**					
Low	Ref.	Ref.	Ref.	Ref.	Ref.
High	0.93 (0.8,1.07)	0.93 (0.8,1.07)	0.93 (0.8,1.07)	0.93 (0.8,1.07)	0.93 (0.8,1.07)
Body mass index (BMI)*Gender					
Adolescent boys^a^ Not overweight/obese		Ref.			
Adolescent boys^a^ Overweight/obese		1.18 (0.78,1.79)			
Adolescent girls ^a^ Not overweight/obese		0.72*(0.62,0.83)			
Adolescent girls ^a^ Overweight/obese		0.74 (0.48,1.15)			
Abdominal obesity*Gender					
Adolescent boys ^a^ No			Ref.		
Adolescent boys^a^ Yes			1.26 (0.74,2.17)		
Adolescent girls ^a^ No			0.73*(0.63,0.84)		
Adolescent girls ^a^ Yes			0.41 (0.16,1.07)		
Triceps skinfold thickness (TSFT)*Gender					
Adolescent boys^a^ Not overweight				Ref.	
Adolescent boys^a^ Overweight				0.96 (0.63,1.45)	
Adolescent girls ^a^ Not overweight				0.72*(0.62,0.83)	
Adolescent girls ^a^ Overweight				0.48 (0.21,1.1)	
Subscapular skinfold thickness (SSFT)*Gender					
Adolescent boys^a^ Not overweight					Ref.
Adolescent boys^a^ Overweight					2.23*(1.53,3.23)
Adolescent girls ^a^ Not overweight					0.72*(0.62,0.84)
Adolescent girls ^a^ Overweight					1.38 (0.9,2.11)

^a^ Interaction; *OR:* Odds Ratio; *CI:* Confidence Interval; *Ref:* Reference; *if *p* < 0.05

Note: All the models have been run after controlling the other socio-demographic factors

*Model 2* represents the interaction between body mass index and gender for pre-diabetes/diabetes. Interestingly, adolescent girls with not overweight/obese category were significantly less likely to suffer (OR, 0.72; CI: 0.62–0.83) from pre-diabetes/diabetes compared to adolescent boys who belonged to the not overweight/obese category. Interaction between gender and abdominal obesity for pre-diabetes/diabetes condition is presented in *Model 3*. Adolescent boys having abdominal obesity were more likely (OR, 1.26; CI: 0.74–2.17) to suffer from pre-diabetes/diabetes than adolescent boys with no abdominal obesity. Moreover, adolescent girls with abdominal obesity were significantly less likely (OR, 0.73; CI: 0.63–0.84) to suffer from pre-diabetes/diabetes compared to adolescent boys with no abdominal obesity. Results from *Model 4* revealed the interaction between gender and triceps skinfold thickness (TSFT) for pre-diabetes/diabetes among adolescents. The likelihood of pre-diabetes/diabetes among overweight (TSFT) adolescent boys was lower (OR, 0.96; CI: 0.63–1.45) than a not overweight adolescent boys. However, the chances of pre-diabetes/diabetes among girls with not overweight category were significantly lower (OR, 0.72; CI: 0.62–0.83) compared to boys with no overweight category. *Model 5* shows the interaction between gender and subscapular skinfold thickness (SSFT) for pre-diabetes/diabetes among adolescents. SSFT had a significant effect on diabetes among both adolescent boys and girls. The probability of getting diabetes was significantly higher (OR, 2.23; CI: 1.53–3.23) among overweight (SSFT) adolescent boys than those who were not overweight. Moreover, adolescent girls from the not overweight category were significantly less likely (OR, 0.72; CI: 0.62–0.84) to suffer from pre-diabetes/diabetes compared to boys from the not overweight category.

## Discussion

The overall prevalence of pre-diabetes/diabetes was 12.3% and 8.4% among adolescent boys and girls in India respectively. Moreover, it was found that body mass index (BMI) and subscapular skinfold thickness (SSFT) was significantly associated with pre-diabetes/diabetes among adolescents in India. Additionally, it was revealed that adolescent girls were less likely to suffer from pre-diabetes/diabetes than adolescent boys in every category of obesity measure.

Earlier studies found that no accessibility of proper care such as early diagnosis, diabetes care management, and blood glucose testing available in primary healthcare settings in the early phases of life for children and adolescents [16, 37]. Early diagnosis of pre-diabetes/diabetes can have lesser risk in the long-term than remaining undiagnosed.

The current study reveals the fact that there was a positive association between BMI, SSFT, and low physical activity status with pre-diabetes/diabetes conditions among adolescents in India. The results were consistent with previous findings that increased BMI was positively associated with pre-diabetes/diabetes conditions among adolescents [20]. Moreover, SSFT was also a significant risk factor of pre-diabetes/diabetes conditions among adolescents, as argued by previous literature [38–40]. Apart from obesity, genetic and metabolic factors, there are other significant risk factors, but in the present study, those were not taken into account due to data limitations [2, 26, 37]. Additionally, low physical activity status was found to be a substantial risk factor for pre-diabetes/diabetes conditions among adolescents, as found in previous studies [41]. It was too argued that even a moderate level of physical activity could help in the prevention of pre-diabetes conditions by reducing obesity levels among adolescents [41].

Dietary practices with unhealthy food such as high intake of junk foods and sugar-sweetened beverages are associated with a high prevalence of obesity/overweight that may lead to pre-diabetes/diabetes conditions among adolescents [42, 43]. This finding is consistent with findings of the present that adolescents with junk food consumption frequency of 3–7 days a week had a high prevalence of pre-diabetes/diabetes condition. Additionally, the menace of substance use among adolescents was a staunch factor for pre-diabetes/diabetes conditions. The finding was paralleled with the previous study that abnormal alcohol and drug use among adolescents increases the risk of pre-diabetes/diabetes conditions [44].

Although it was earlier argued that diabetes is an affluent disease and it occurs among the population from higher socio-economic strata [16], however, the present study revealed that the disease pattern is now shifting towards lower affluent population, for instance, pre-diabetes/diabetes condition was more among SC/ST, poor and adolescents from rural areas especially in case of adolescent girls. Further research is needed to look into the highly prevailing pre-diabetes/diabetes among adolescents irrespective of caste, class, gender and geographical locations.

The results showed that pre-diabetes/diabetes vary across gender; for instance, gender differential was over 4%, i.e., adolescent boys are more prone to pre-diabetes/diabetes than adolescent girls. Moreover, for most of the background factors, adolescent boys were at high risk than adolescent girls. Even previous literature argued that adolescent girls do have a lower probability to suffer from pre-diabetes/diabetes [45]**.** The plausible reasons can be derived through descriptive statistics in the present study where it was clearly visible that obesity levels and substance use were low among adolescent girls and which may further protect them from the burden of pre-diabetes and diabetes condition in comparison to their counterpart. Additionally, interaction models revealed that adolescent girls were less likely to suffer from pre-diabetes/diabetes in comparison to adolescent boys for the obesity measure. The findings are found to be consistent with the previous one as it was argued that adolescent girls are less likely to be overweight or obese [46].

### Limitation

The study has potential limitations. This study used cross-sectional data that does not allow establishing a causal relationship between variables. Additionally, Type 1 Diabetes mellitus, which is more common in adolescents, is not taken into account which is also a major limitation of this article. Glucose Tolerance Test, Fasting Blood Sugar (FBS) test and Post Prandial Blood Sugar (PPBS) test were not performed. Despite the limitation, the study also has its own strength. This study used large scale survey data of adolescents which is the first-ever survey on nutrition and gives reliable estimates on diabetes at the national level.

## Conclusion

Our study found that pre-diabetes/diabetes was highly prevalent among adolescents especially among adolescent boys in India. Interaction models in the present study clearly reveal the fact that adolescent girls are less likely to suffer from pre-diabetes/diabetes than adolescent boys. BMI and SSFT are the two most important predictors of pre-diabetes/diabetes among adolescents in India. The high prevalence of pre-diabetes/diabetes among adolescent girls from lower socio-economic strata poses a high concern in the Indian scenario. The emerging type 1 diabetes (T1D) among children and adolescents cannot be prevented with existing knowledge [3]. However, type 2 diabetes can be prevented with effective approaches available, and premature deaths can be curtailed off [3]. To avoid diabetes and related risk factors, regular exercise, maintaining a healthy diet, avoiding substance use, and controlling blood pressure and lipids as recommended by WHO guidelines [3]. The present study also suggests that to prevent pre-diabetes/diabetes conditions among adolescents certain health interventions are required. Utilization of knowledge, awareness, and programme (KAP) re-intervention to control obese/overweight among adolescents on the one hand and the regular health check-ups among adolescents, on the other hand, will surely help to curtail pre-diabetes/diabetes conditions among adolescents in India. Adolescent sub-populations are out of the coverage of the special programme that addresses the crucial public health issues of diabetes and related various risk factors.

## Data Availability

We have provided details of the data in the methodology section. The CNNS data can be obtained on request from the Population Council, Delhi (India). The report and the survey tools are also available on the website: https://www.popcouncil.org/uploads/pdfs/2019RH_CNNSreport.pdf and cnns.pc@gmail.com respectively.

## Abbreviations

MoHFW: Ministry of Health and Family Welfare
UNICEF: United Nations Children Funds
CNNS: Comprehensive National Nutrition Survey
BMI: Body Mass Index
HbA1c: Glycosylated haemoglobin
T1D: Type-1 diabetes
T2D: Type-2 diabetes
GDM: Gestational diabetes mellitus
TSFT: Triceps skinfold thickness
SSFT: Subscapular skinfold thickness
LDL: Low-density lipoprotein
HDL: High-density lipoprotein
SC/ST: Scheduled Tribe/Scheduled Caste
